# Investigation of The Hepatoprotective Potential of Liposomal Resveratrol as Polyphenols Against Liver Damage in Streptozotocin Diabetic Rat Model

**DOI:** 10.7150/ijms.109115

**Published:** 2025-07-24

**Authors:** Ahmed Z. Alanazi, Mohammad M. Algahtani, Faisal A. Alotaibi, Salim S. Al-Rejaie, Mohammed Alqinyah, Abdullah S. Alhamed, Hussain N. Alhamami, Ahmed Nadeem, Mohammad Raish, Khaldoon Aljerian, Adel M. Alragas, Khalid Alhazzani

**Affiliations:** 1 Department of Pharmacology and Toxicology, College of Pharmacy, King Saud University, Riyadh, Saudi Arabia.; 2 Department of Pharmaceutics, College of Pharmacy, King Saud University, Riyadh, Saudi Arabia.; 3 Department of Pathology, College of Medicine, King Saud University, Riyadh, Saudi Arabia.; 4 Department of Clinical Pharmacy, College of Pharmacy, King Saud University, Riyadh, Saudi Arabia.

**Keywords:** diabetes, liposomal resveratrol, hepatic failure, inflammation, oxidative stress

## Abstract

Diabetes Mellitus is a prominent contributor to degenerative diseases globally and is often associated with hepatic injury. The dysfunction of the liver is characterized by cirrhosis, inflammation, apoptosis, and impaired antioxidant defense mechanisms. Our goal was to estimate the hepato-protecting properties of Liposomal resveratrol (LR) administered at 20 and 40 mg/kg in diabetic rat models and compare them with those of resveratrol at 40 mg/kg. The diabetes was induced in them using streptozotocin (STZ) (65 mg/kg) during a five-week long dosage. This study investigates the effects of LR and resveratrol on glucose levels, body weight, inflammatory markers (TNF-α, IL-6, NF-κB), oxidative stress parameters (MDA, catalase, GPx), apoptotic markers (caspase-3, BAX, BCL-2), liver function enzymes (SGOT, SGPT, GGT), and histopathological alterations in liver tissue. Diabetic rats infused with STZ exhibit changes in hepatic function markers, and increased inflammatory and apoptotic responses, all of which were reversed by administering LR. This reversal occurred through the downregulation of TNF-α and IL-6, inhibition of NF-κB translocation, upregulation of BCL-2, and downregulation of caspase 3 and Bax protein levels. STZ-induced diabetic rats experience a significant disruption in their antioxidant defense system, whereas LR administration notably inhibits lipid peroxidation and significantly enhances the activity of antioxidant enzymes. The histopathological analysis of liver tissue in STZ rats showed morphological changes when compared to the normal rats. This was indicative of the development of acute inflammations. In contrast, LR treatment resulted in normal liver histology with minimal presence of chronic inflammatory cells, predominantly lymphocytes. Remarkably, resveratrol alone was less effective than LR in restoring these parameters in diabetic subjects. Administration of LR effectively mitigates oxidative stress and hepatic impairment caused due to diabetes, suggesting its potential as a therapeutic agent to reduce liver dysfunction in diabetic patients.

## 1. Introduction

Diabetes Mellitus (DM), a prevalent metabolic disorder, is characterized by chronic hyperglycemia due to abnormalities in insulin secretion, insulin action, or both 1, 2. It is a significant global health concern, associated with various complications that occur in both type 1 and type 2 diabetes, such as nephropathy, neuropathy, delayed wound healing, retinopathy, cardiovascular disease, and liver dysfunction. Among these, liver disease is particularly notable due to the liver's critical role in eliminating harmful substances. However, its vulnerability to oxidative stress and other factors makes it a target for dysfunction 3.

The liver, being the largest, most important, and highly complex internal organ of the body, can eliminate harmful substances. Despite its beneficial functions, the liver is vulnerable to various factors that can interfere with its functioning, of which oxidative stress is a notable concern 3. DM can cause severe liver damage, known as diabetic-associated hepatotoxicity since the liver is essential for the metabolism of glucose 4. Diabetes-associated hepatotoxicity is a significant complication of DM 5. Patients with diabetes have a higher chance of developing multiple liver diseases, including cirrhosis, fibrosis, viral hepatitis, hepatocellular carcinoma, and non-alcoholic fatty liver disease 6. Inflammation and apoptosis in the liver are primarily triggered by the release of inflammatory cytokines and the activation of the NF-κB pathway. Additionally, an imbalance in apoptotic markers such as caspase 3, BAX, and BCL-2 contributes significantly to liver damage by disrupting the regulation of cell death and survival 7. Therefore, the attenuation of hyperglycemia, along with its associated oxidative stress and inflammatory responses, is considered a viable strategy for mitigating liver damage induced by diabetes 8.

Notably, oxidative stress is one factor that stands out among numerous other factors in the advancement of disease and disorders. It is considered an important factor driving a variety of diseases. Liver damage is one of the conditions associated with oxidative stress, especially in individuals with diabetes. Another factor that co-occurs in almost all diseases is the elevation in reactive oxygen species (ROS) levels specifically in individuals with diabetes. It disrupts the balance between these species and the body's antioxidant defense mechanism. This imbalance is one of the reasons for driving oxidative stress which as a result causes damage at cellular and tissue levels through processes such as lipid peroxidation, protein oxidation, and DNA breakage 2, 9. These mechanisms can significantly affect liver function and cause inflammation, fibrosis, and apoptosis. In brief, the onset of inflammation and apoptosis in the liver is primarily attributed to the secretion of inflammatory cytokines, as well as the activation of the proinflammatory gene regulator known as nuclear factor kappa B. Furthermore, findings from research studies indicate that dysregulation in the apoptotic markers' expression such as caspase 3, BAX, and BCL-2 also play significant roles in this process 7, 10. Also, various studies have showed that providing the dosage of STZ markedly enhances the expression of regulators associated with inflammation, apoptosis, and oxidative stress 11-14. Numerous studies provide strong evidence that administering STZ induces diabetes mellitus and leads to the subsequent expression of regulators associated with inflammation, apoptosis, and oxidative stress, making it a widely used experimental model for diabetes research 11-14.

STZ induces diabetes primarily through selective pancreatic β-cell toxicity, mediated by its uptake via the GLUT2 glucose transporter, which is highly expressed in β-cells 15, 16. Once inside, STZ causes DNA alkylation and strand breaks, activating poly(ADP-ribose) polymerase (PARP), which depletes intracellular NAD⁺ and ATP, leading to β-cell necrosis 15, 16. Furthermore, STZ generates reactive oxygen species (ROS) and nitric oxide (NO), resulting in oxidative stress, to which β-cells are particularly susceptible due to their low antioxidant capacity 15, 16. In addition, STZ induces an inflammatory response, increasing the expression of cytokines such as IL-1β and TNF-α, which further promote β-cell damage through NF-κB activation and apoptotic pathways 15, 16. In our model, a single intraperitoneal dose of 65 mg/kg was used to induce Type 1 diabetes via direct cytotoxicity and irreversible β-cell destruction, consistent with previous literature 17, 18.

The historical use of natural phytochemicals for diabetes treatment is gaining wider acceptance due to their robust anti-disease benefits and minimal side effects compared to alternative therapies. Resveratrol, a polyphenolic compound that belongs to the stilbenoid family, is found in abundance in berries and alike fruits. The distinctive and unique chemical structure of resveratrol facilitates the delocalization of electrons, thereby improving its ability to scavenge radicals. It comprises two phenolic rings, which facilitate in H atoms' sharing. Additionally, it contains three OH groups which enhances its ability to counteract oxidative stress and chelating metal ions, thereby inhibiting ROS production 19. Research outcomes display important outcomes over the past several years to validate the human health benefits of resveratrol through experimental studies, which have revealed its ability to reduce oxidative damage, and apoptosis in disease models 20-24.

Moreover, resveratrol diminishes malondialdehyde (MDA) levels, affirming its antioxidant efficacy. Although previous evidence has shown the ability of resveratrol to combat diabetes and associated complications in animal models, clinical studies have yielded varying findings which may be due to variations in dosage and absorption rates 25. Therefore, to address the challenge of limited bioavailability and enhance effectiveness, it is crucial to explore alternative strategies that not only improve absorption and bioavailability but also enable targeted drug delivery. In an attempt to validate the efficacy of LR, Gines and colleagues conducted a study in 2017, showing that LR efficiently diminished pancreatic damage in SAMP8 mice by suppressing inflammatory cytokines, mitigating oxidative stress, and apoptosis, along with the increase in SIRT1 expression 26. In a related study, Sannigrahi and the team sought to examine the impact of LR in rats with carbon tetrachloride-induced hepatic destruction. The results showed that administering resveratrol liposome doses (100 mg/kg and 200 mg/kg) over an 8-week period efficiently lowered the levels of oxidative stress, suppressed cell apoptosis, and lowered the expression of proinflammatory cytokines, thus alleviating hepatic damage 27, while the 200 mg/kg dosage showed a more pronounced effect. In addition, liposomes can overcome the pharmacokinetic limitations of resveratrol by making the drug take on the pharmacokinetic characteristics of the carrier. Using liposomes as a drug delivery vehicle becomes a promising strategy to enhance the bioavailability of resveratrol 28. LR has been shown to reduce pancreatic damage in mice and hepatic damage in rats, by suppressing inflammatory cytokines, mitigating oxidative stress, and apoptosis. LR treatment effectively countered cardiac dysfunction and kidney damage through the reduction of oxidative stress, inflammation, and apoptosis in cardiac and kidney tissues of streptozotocin-induced diabetic rats, demonstrating its potential to mitigate diabetes-associated complications 17, 18.

To date, there is no scientific evidence indicating that LR has beneficial effects in halting the progression of diabetes-related liver damage in any study. Therefore, by altering nuclear transcription factors, this research endeavors to explore LR's therapeutic potential as a hepatoprotective agent against diabetic-induced hepatotoxicity in type 1 DM by alleviating inflammation and oxidative stress. In order to effectively reduce the abundance of diabetes and its correlated complications, it is crucial to understand the complex interactions among diabetes, oxidative stress, and liver damage.

## 2. Material and Methods

### 2.1 Chemicals and materials

Resveratrol and STZ were obtained from Sigma-Aldrich, Inc. (St. Louis, MO, USA). Liposomal Trans RES® formulation (~200 nm) was provided by Lipolife® (Drakes Lane, UK). Fasting blood glucose levels were measured using the Accu-Chek Compact-Plus glucose meter system (Roche Diagnostics, Meylan, France). Proinflammatory biomarkers (TNF-α, NF-kB, and IL-6) and enzymatic activities of MDA, CAT, and GPx were determined using ELISA kits from R&D Systems Inc. (Minneapolis, MN, USA). Serum biomarkers, including SGPT, CGT, and SGOT, were measured using commercially available diagnostic kits from Human (Wiesbaden, Germany). A high-capacity cDNA archive kit was obtained from Applied Biosystems (Waltham, MA, USA) while primers were purchased from GenScript (Piscataway, NJ, USA).

### 2.2 Induction of diabetes

After an overnight fast, male Wistar rats were given a single intraperitoneal injection of STZ (65 mg/kg) to induce type 1 diabetes mellitus as previously described 29. Thereafter, we prepared the STZ-containing solution freshly. The buffer used for preparing the STZ dosage was citrate buffer, and the concentration was 0.1 M, maintained at a pH of 4.5. Diabetes occurrence was marked only when the rats used in this study displayed a glucose level of at least 250 mg/dL. Then these rats were picked and used in the subsequent studies.

### 2.3 Animal maintenance conditions

Prior to initiating the diabetic animal study, 30 male Wistar rats were provided on demand by the experimental animal care center, college of pharmacy, KSU upon obtaining the ethical clearance and other necessary formalities. The details of the rats obtained are as follows: age in weeks were 6 to 7, weight ranging from 250 to 270 gm, and the type is male Wistar. Their environment was meticulously regulated. This includes keeping a steady room temperature of 22 ± 1°C and a level of humidity between 50 and 55 %. A half-day cycle of 12 light and 12 hours dark was also part of this methodology. Before we proceeded with our experimental studies, the rats were acclimatized so that they could adjust to the newly shifted laboratory environment. This period lasted for 7 days. All the animals used for research-related procedures adhered to the norms created by NIH (NIH publication No. 80-23;1996). Approval for the animal studies was also obtained from the KSU's research ethics committee. The approval number of the study is (KSU-SE-23-36).

### 2.4 Experimental design

After acclimatization, 5 groups were created each with 6 rats, labelled 1 to 5. The dosage delivered were as follows:

Group 1: Control rats with no STZ injection and administered with vehicle-only.

Group 2: Diabetic rats administered with vehicle only.

Group 3: Diabetic rats administered with 20 mg/kg/day oral LR for 5 weeks starting on day 8. Group 4: Diabetic rats administered with 40 mg/kg/day oral LR for 5 weeks starting on day 8. Group 5: Diabetic rats administered with 40 mg/kg/day oral resveratrol for 5 weeks starting on day 8.

**Figure 1:**
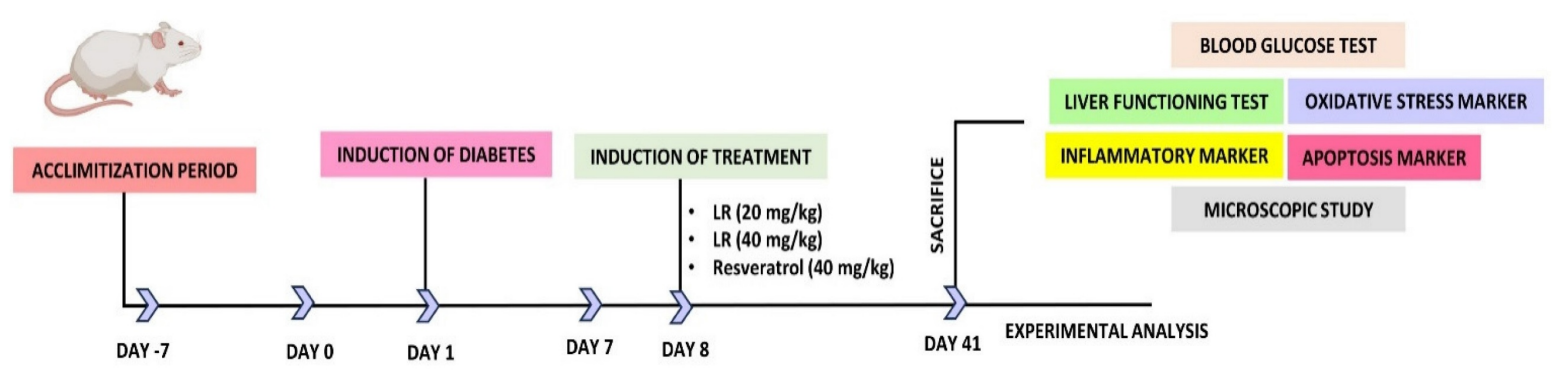
Schematic representation of followed steps for the analysis of LR from the beginning of acclimatization to experimental analysis.

On the 42^nd^ day, the rats were first anesthetized to reduce their suffering and later euthanized. For painless euthanasia, a mixture of two chemicals, xylazine and ketamine was given in the concentrations of (10 mg/kg) and (92 mg/kg). A cardiac puncture was made and then the sample required for centrifugation was collected. Centrifugation was done at a rotation speed of 2000 × g for ten minutes. Subsequently, the separated samples were kept inside the refrigerator at the temperature of -20 °C till further testing. Post-dissection of the liver from each group, a cross-section was placed in 10% formalin. For histological analysis. The rest of the unused liver tissues were promptly preserved at -80 °C using liquid nitrogen. Figure 1 illustrates the dosing schedule and the chart for diabetes induction.

### 2.5 Measuring weight and blood glucose levels

The changes in body weight of rats in each experimental group, beginning from the time of STZ administration, were documented through a precise mini-weighing balance. Along with it the levels of the blood glucose were measured one time per week to determine the effect of LR on diabetic rats.

### 2.6 Biomarkers estimation

Various biomarker levels were measured to determine the functioning level of the liver. These tests include Serum glutamate pyruvate transaminase (SGPT), Gamma glutamyl transferase (CGT), and serum glutamic oxaloacetic transaminase (SGOT). For this step, we ordered and utilized commercially sold diagnostic kits from the manufacturer named Human (Wiesbaden, Germany). Low as well as higher doses were examined using the mentioned kit to make a comparison between low and higher dosage amounts with respect to resveratrol in the absence of any dose.

### 2.7 Biomolecular profiling

Our next objective was to measure the levels of factors involved in transcription, oxidative stress markers, and inflammatory mediators. This investigation was carried out using specific procedures, as diabetes is known to elevate these factors. Proteinase inhibitors were incorporated into chilled phosphate-buffered saline (PBS) in order to the homogenization of liver samples, at a concentration of 10% w/v. Subsequently, centrifugation of the homogenate yielded a transparent, clear supernatant fraction. The protein content of this supernatant fraction was quantified utilizing Bradford's reagent. Following this, ELISA kits (obtained from R&D Systems Inc., Minneapolis, MN, USA), were utilized to measure the amounts of NF-kB, TNF-α, and IL-6. This quantification was conducted in accordance with the manufacturer's guidelines.

#### 2.7.1 Assessment of antioxidant activities

The oxidative stress process leads to the formation of malondialdehyde (MDA) and its expression increases under hyperglycemic conditions. To measure the levels of TBARS (thiobarbituric acid reactive substances), we used a diagnostic kit purchased from Cayman Chemical Company, USA, following the manufacturer's instructions.

In addition to MDA measurements, we also analyzed the activities of Catalase (CAT) and Glutathione peroxidase (GPx) under diabetic conditions. To achieve this, we collected liver post-mitochondrial supernatants for the estimation of CAT and GPx enzymatic activities. These assessments were performed using assay kits obtained from R&D Systems Inc., USA, following the manufacturer's instructions.

### 2.8 RT PCR

Subsequently, it was imperative to analyze the mRNA manifestation of several apoptotic markers, containing Caspase 3, BAX, and BCL-2, within liver tissues. High-capacity cDNA archive kit was purchased (from Applied Biosystems, USA) which was used for aiding the process of reverse transcription. After obtaining the primers (from the GenScript company, Piscataway, NJ, USA) the ABI PRISM 7500 Sequence Detection System (from Applied Biosystems) was occupied to assess the gene expression of these apoptotic markers. Beta-actin (β-actin) was employed as the housekeeping gene for normalization. Relative quantification was estimated using the 2-^ΔΔCt^ method as follows:

ΔCt (control)= Ct (target gene, control sample) - Ct (β-actin; control sample)

ΔCt (treatment) = Ct (target gene, treatment sample) - Ct (β-actin; treatment sample)

ΔΔCt = ΔCt (treatment) - ΔCt (control)

Then fold-change expressed as 2-^ΔΔCt^ ± Standard deviation (S.D).

### 2.9 Histopathological analysis

The histopathological analysis of the liver section was performed. This is done to evaluate liver histology and the limits of inflammatory response in rats undergoing LR therapy. This evaluation utilizes the Leica CM3050S research cryostat (manufactured by Leica Bio-Systems in the United States), to section, embed in paraffin wax, and slice the samples of liver tissues in 5 μm thick sectioning. The sliced tissue was subsequently stained using the stains Hematoxylin and Eosin dyes and prepared for inspection through light microscopy to observe any liver damage resulting from the treatment. The analysis was performed by a professional histopathologist. The Suzuki score was employed to quantitatively assess the extent of liver damage, focusing on parameters such as congestion, hepatocyte necrosis, and vacuolization. Each parameter was scored on a scale from 0 to 4, where 0 indicates no damage, 1 indicates minimal damage, 2 indicates mild damage, 3 indicates moderate damage, and 4 indicates severe damage.

### 2.10 Statistical examination

In this study, we assessed the differences between groups by comparing the mean values and standard errors using two tests, (which are Student-Newman-Keuls multiple comparison test and one-way ANOVA). Statistical significance was assessed using p-values, with significance levels denoted as follows: *p < 0.05, **p < 0.01, and ***p < 0.001. A common Software was used for this purpose (GraphPad Prism 9).

## 3. Results

### 3.1 Administration of LR attenuates blood glucose levels and body weight loss

**Figure 2:**
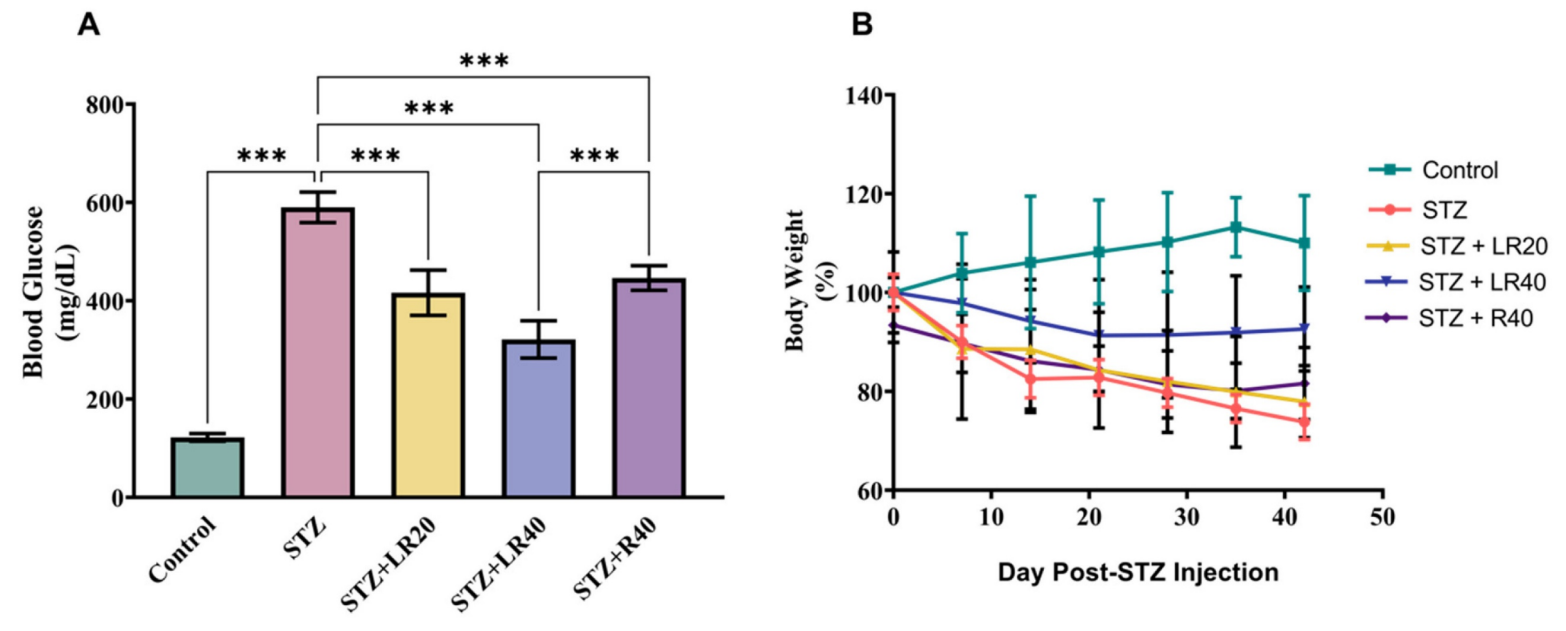
The impact of LR dosages on: A) Glucose levels, B) Body weight (Mean ± Standard Error. Substantial variations are denoted as ***p < 0.001 compared to the control group, ***p < 0.001, **p < 0.01, and *p < 0.05 when compared to the STZ induced diabetic group).

Figure 2A presents a detailed analysis of glucose levels. Diabetic rats experienced a significant 2.75-fold rise in total the total levels of glucose (p < 0.001), when evaluated against the healthy ones in the controlled group. However, treating them with LR at the concentration of 20 mg/kg and the increased concentration of 40 mg/kg resulted in noteworthy decreases in glucose levels, with respective reductions of 1.8-folds (p < 0.001) and 2-folds (p < 0.001). Figure 2B depicts the body weight changes in diabetic rats over time subsequent to STZ treatment. The data suggests that non-diabetic rats fed a normal pellet diet had higher body weights, whereas those infused with STZ showed a noticeably higher decline in body mass. Moreover, administering LR at 20 mg/kg and 40 mg/kg to STZ-treated diabetic rats led to substantial improvements in body weight. Interestingly, when juxtaposed with the group receiving the 20 mg/kg dosage, the group administered the 40 mg/kg dose of LR showed the most significant enhancement in body weight. In addition, the 40 mg/kg dose of resveratrol significantly reduced blood glucose levels, while no notable effect was observed on body weight.

### 3.2 Administration of LR attenuates hepatic inflammation in hepatic tissues of diabetic rats infused with STZ

**Figure 3:**
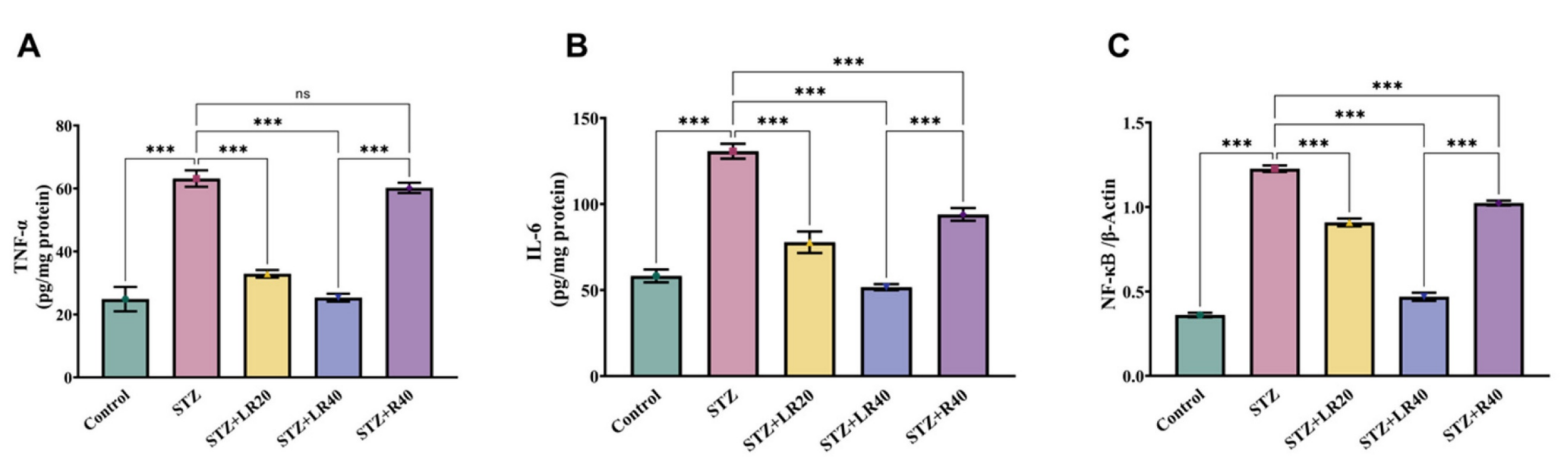
This figure displays the comparison data of the following: A) TNF-α, (B) IL-6, and (C) NF-kB within liver tissues. The units of concentrations used here for proteins are as pg/mg (where, mean ± Standard Error. Variations are denoted as ***p < 0.001 compared to the control group, ***p < 0.001, **p < 0.01, and *p < 0.05 when compared to the STZ induced diabetic group).

Since inflammation-associated genes are overexpressed in diabetes, we investigated whether treatment with LR and resveratrol could modulate their expression levels in diabetic rats. As depicted in Figure 3A-C, the levels of expression of TNF-alpha (2.5 times; p < 0.001), IL-6 (2.2 times; p < 0.001), and NF-κB (3.4 times; p < 0.001) was elevated in diabetic subjects compared to the vehicle control group. This indicates that the levels of inflammatory genes are upregulated under diabetic conditions. Furthermore, as compared to STZ-induced diabetic subjects, giving the dosage of LR at 20 mg/kg and 40 mg/kg notably decreased the levels of such inflammatory-causing mediators. Notably, the 40 mg/kg dose of LR exhibited more pronounced effects, resulting in a respective reduction of TNF-alpha by 2.5-fold, IL-6 by 2.5-fold, and NF-κB by 2.6-fold. Similarly, 40 mg/kg dosage of resveratrol showed a significant decrease in the expression of TNF-alpha (1.04 times; p < 0.01), IL-6 (1.3 times; p < 0.001), and NF-κB (1.2 times; p < 0.001) markers. These results confirm the superior protective impact of LR therapy in reducing hepatic inflammatory damage in diabetic rats through the downregulation of inflammatory mediators, at both dosages.

### 3.3 Administration of LR attenuates oxidative stress in hepatic tissues of diabetic rats infused with STZ

**Figure 4:**
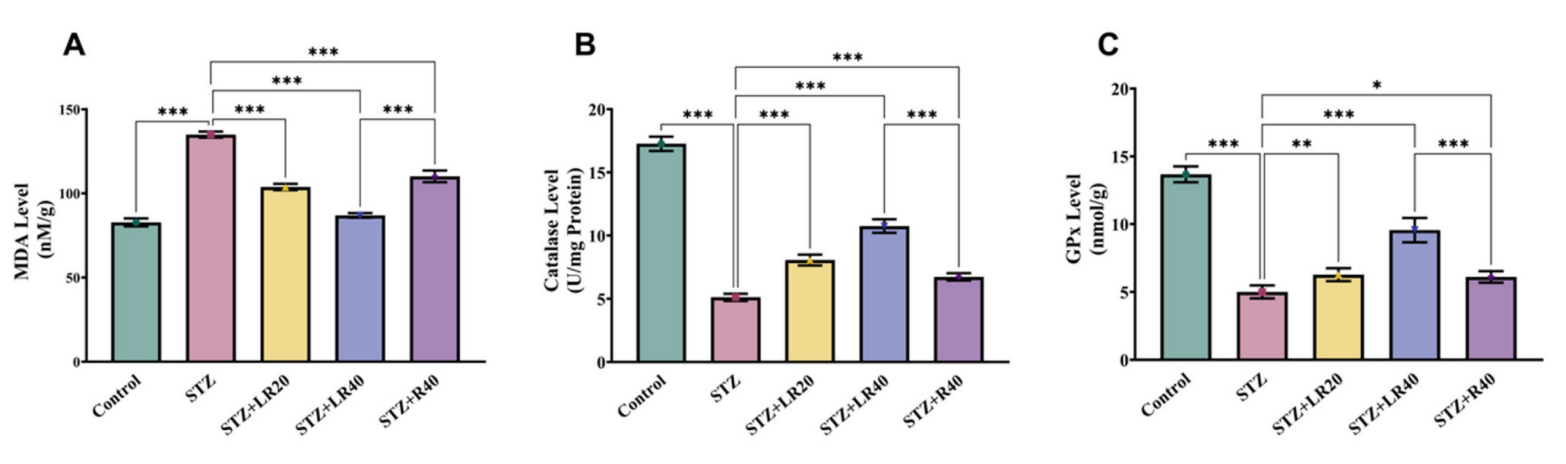
The figure presents bar graphs showing the effects of LR (20 and 40 mg/kg) and resveratrol (40 mg/kg) treatments on liver antioxidant levels in rats across experimental groups. The graphs depict (A) lipid peroxidation (n moles of MDA/mg protein), (B) Catalase activity (units/mg protein), and (C) GPx activity (units/mg protein). Data are expressed as Mean ± Standard Error, with statistical significance denoted by ***p < 0.001 compared to the control group and ***p < 0.001, **p < 0.01 compared to the STZ group. NS indicates non-significant differences. ​

After analyzing the protective effects of LR against inflammation in liver tissues of diabetic rats, we examined the inhibitory consequences of LR at both doses on MDA production, a marker of oxidative stress. As depicted in Figure 4A, the liver tissues of the subjects treated with STZ exhibited a rise in lipid peroxidative damage (1.6 times; p < 0.001), in contrast with the vehicle group. Furthermore, administering LR at 20 mg/kg and 40 mg/kg remarkably declined lipid peroxidation levels of diabetic subjects, with the 40 mg/kg dose exhibiting stronger effects, resulting in a 1.5-fold reduction. Similarly, the resveratrol-administered group with a concentration of 40 mg/kg dosage showed a significant decrease in the expression of malondialdehyde (1.2-fold; p < 0.001), compared to the STZ group. In conclusion, such insights imply that administering LR at a high dose significantly reduces lipid peroxidation associated with STZ-induced diabetes by lowering the expression of MDA.

Furthermore, we assessed the antioxidant potential of LR on a panel of oxidative stress indicators.

This is done through the evaluation of the hepatic enzymatic activities of proteins including catalase and glutathione peroxidase. As illustrated in Figures 4B and 4C, levels of these indicators were higher in the control group, while diabetic ones showed a significant decrease in these enzyme levels, by 3.3 and 2.7 times. Notably, administering LR at 20 mg/kg and 40 mg/kg displayed a dosage-driven outcome, with the concentration of 40 mg/kg, producing more pronounced effects, leading to a 2.1-fold and 1.9-fold rise in the activity of these enzymes. However, treatment with 40 mg/kg resveratrol partially restored the activity of these enzymes in diabetic ones but less effective than LR.

### 3.4 Administration of LR Inhibits apoptosis in hepatic tissues of diabetic rats infused with STZ

To evaluate the protective effect of LR on the regulation of apoptosis markers in liver tissues of STZ-infected diabetic rats, real-time PCR analysis was conducted.

**Figure 5:**
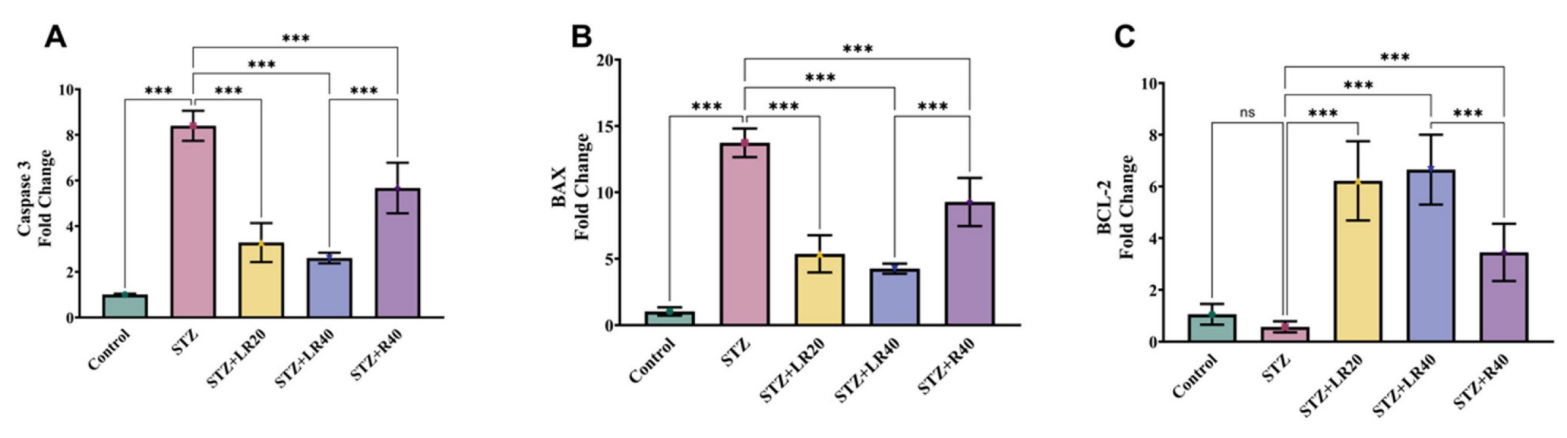
The figure displays bar graphs representing the expression levels of (A) Caspase 3, (B) BAX, and (C) BCL-2 in liver homogenates, quantified using real-time PCR. Data are presented as Mean ± Standard Error, with statistical significance indicated by ***p < 0.001 compared to the control group and ***p < 0.001 compared to the STZ group. NS denotes non-significant differences.

As indicated in Figures 5A-C, the normal control group exhibited decreased expression of caspase 3 and BAX, along with increased expression of BCL-2 in liver tissues, suggesting no liver damage. In contrast, diabetic rats infused with STZ showed clear signs of liver damage, evidenced by a significant elevation in the level of caspase 3 and BAX by 8.3-fold and 13.7-fold, respectively, while the 1.8-fold decrease in BCL-2 levels was not significant compared to the control group. Contrarily, treatment with LR at 20 mg/kg and 40 mg/kg restored these parameters, demonstrated by the decline in the level of caspase 3 by 2.5-fold and 3.2-fold, and BAX by 2.5-fold and 3.2-fold, along with an increase in BCL-2 levels by 10.8-fold and 11.6-fold. Likewise, administering resveratrol at a dosage of 40 mg/kg exhibited slightly less impact on these apoptotic markers, compared to LR-treated diabetic rats. This was evidenced by a decrease in caspase 3 levels by 1.4-fold and BAX levels by 1.4-fold, coupled with an increase in BCL-2 levels by 6-fold. Nonetheless, treatment with resveratrol alone at 40 mg/kg demonstrated significant inhibition of these apoptotic markers compared to the untreated diabetic group. The findings strongly proffer that treatment with both doses of LR and resveratrol alone can notably improve hepatic function in diabetic animals by effectively inhibiting apoptosis.

### 3.5 Administration of LR enhances liver enzymatic functions in hepatic tissues of diabetic rats infused with STZ

**Figure 6:**
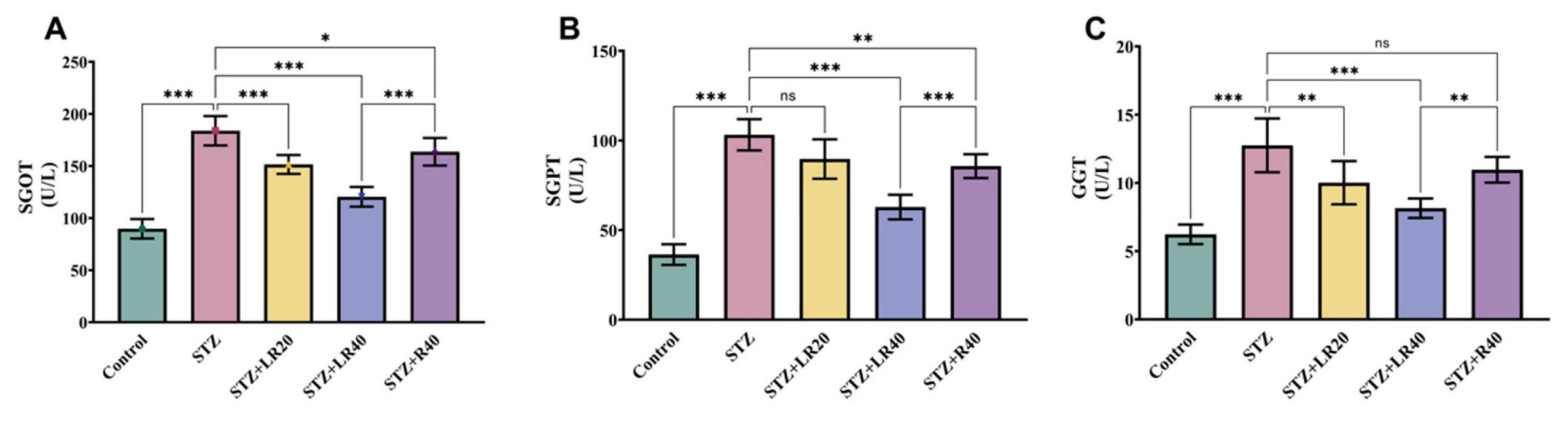
The figure here shows the impact of LR (20 and 40 mg/kg) and resveratrol (40 mg/kg) treatments on the serum concentrations of (A) SGOT, (B) SGPT, and (C) GGT in diabetic rats (n=6). The presented data are expressed as Mean ± Standard Error, with statistical significance denoted as ***p < 0.001 relative to the control cohort, and ***p < 0.001, **p < 0.01, *p < 0.05 relative to the STZ group. NS signifies non-significant variations.

The results related to liver enzymes are shown in Figure 6A-C. As expected, diabetic rats demonstrated a significant increase in SGPT (2.8 times; p < 0.001), SGOT (2 times; p < 0.001), and GGT (2-fold; p < 0.001) relative to the control cohort (p < 0.001). But, after administering LR at 20 mg/kg and 40 mg/kg to diabetic rats, we were able to observe a substantial decrease in SGPT (by 1.1-fold and 1.6-fold), SGOT (by 1.2-fold and 1.5-fold), and GGT (by 1.2-fold and 1.5-fold) expression levels. Remarkably, the administration of resveratrol at a dose of 40 mg/kg engendered a significant reduction in the quotients of these markers (by 1.2-fold; p < 0.01, 1.1-fold; p < 0.001, 1.1-fold; p < 0.01), when compared to untreated STZ-infused diabetic rats. These findings collectively confirm that LR, at both dosages, effectively improves liver enzyme function by decreasing their expression in STZ-induced diabetic animals.

### 3.6 Administration of LR mitigates liver damage in diabetic rats: Histological analysis

**Figure 7:**
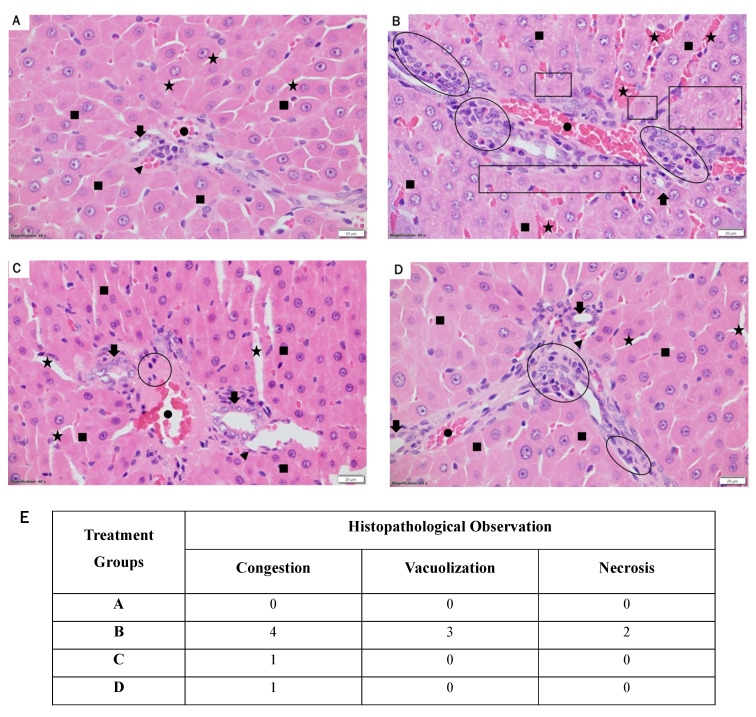
Histopathological alterations in liver sections (60X magnification) are depicted. Liver sections from (A) control rats, (B) streptozotocin-induced diabetic rats, (C) LR-treated rats (40 mg/kg), and (D) resveratrol-treated rats (40 mg/kg) were stained with hematoxylin and eosin and evaluated for the severity of hepatitis. (E) The Suzuki score for the histopathological images to evaluate the degree of liver damage: 0 indicates no damage, 1 indicates minimal damage, 2 indicates mild damage, 3 indicates moderate damage, and 4 indicates severe damage. The photomicrographs represent tissue pathology observed in six animals (n = 6) from each experimental group.​The following shapes are used in the histopathological images: Dot indicates portal venules; stars represent sinusoids; arrowheads denote arterioles; arrows indicate branches of bile ducts; filled squares represent hepatocytes; empty circles/ovals indicate inflammation; and empty squares/rectangles denote necrosis and vacuolation.

A histological investigation was undertaken using light microscopy to assess the impact of LR treatments at two varying doses on the livers of rats with STZ-induced diabetes. Figure 7 presents the representative liver histopathology of the experimental groups as observed under light microscopy. Figure 7 showcases the illustrative liver tissue pathology across the treatment cohorts, as visualized through light microscopy. The liver sections derived from the control group exhibited normal architecture with minimal chronic inflammatory infiltrates, such as lymphocytes, as shown in Figure 7A. The rats induced with STZ-induced diabetes (Figure 7B) exhibited extensive liver damage, marked by severe acute and chronic inflammation (hepatitis), along with notable infiltration of neutrophils, lymphocytes, and plasma cells. Although treatment with resveratrol at 40 mg/kg showed a mild to moderate improvement in hepatitis with decreased inflammation infiltration (Figure 7D), a considerable reversal in the histological irregularities induced by STZ was notably improved with the higher LR dosage (40 mg/kg) (Figure 7C). These alterations encompass a decrease in the incidence of neutrophil and lymphocyte hepatitis. Therefore, these findings collectively emphasize that LR significantly mitigated the hepatic alterations induced by STZ administration.

## Discussion

Diabetes mellitus is a chronic metabolic condition characterized by impaired insulin production or utilization, leading to dysregulation of blood glucose levels 1. This multifactorial disorder manifests due to either insufficient insulin secretion from pancreatic beta cells or inadequate responsiveness of target tissues to insulin action. Since the liver is essential for glucose metabolism, there is a close relationship between liver disease and hyperglycemia 6. Nevertheless, regulatory processes such as glucose metabolism are disturbed in conditions such as cirrhosis, hepatitis, or fatty liver disease 6. These disruptions can lead to alterations in glucose production and insulin responsiveness, ultimately resulting in hyperglycemia or elevated blood glucose levels. Liver damage caused by hyperglycemia has been correlated with heightened production of reactive oxygen species and an abnormal inflammatory response 6. Consequently, individuals with diabetes exhibit a high prevalence of liver dysfunction 30. Most medical treatments for diabetic liver damage are unable to reverse or halt its progression, and current therapeutic approaches remain ineffective. As a result, there is a global search for effective alternatives that can potentially prevent or slow the progression of such conditions. Researchers globally are continuously striving to discover novel phytochemicals that possess the potential not only to decelerate disease progression but also to alleviate associated side effects.

Extensive research conducted to date has demonstrated the capacity of resveratrol to mitigate liver damage induced by high glucose levels in cell lines or animal models. As an example, Chang *et al.* demonstrated that the administration of resveratrol doses of 0.1 mg/kg and 1 mg/kg over a span of seven successive days led to a notable decrease in oxidative stress and inflammatory indicators. This involved a reduction in the concentrations of superoxide anion, protein carbonyl, Mn-SOD, NF-kB, and IL-1β. These results further validate the protective effects of resveratrol on the liver and its anti-inflammatory properties in alleviating liver damage in diabetic animals 29. In a separate investigation, administering oral resveratrol treatment at 5 mg/kg for over 30 days led to reductions in both reductions in glycated hemoglobin concentrations and blood glucose in diabetic rats induced by STZ-nicotinamide. This was followed by a pronounced rise in plasma insulin concentrations. Furthermore, these rats exhibited altered functioning of various key enzymes that aid in the process of metabolization of carbohydrates. Examples of such enzymes are hexokinase, glucose-6-phosphatase, and fructose-1,6-bisphosphate, as well as glycogen synthase in the liver and kidney. However, the administration of resveratrol improved the activity of these enzymes, demonstrating its potential to ameliorate diabetic conditions and exhibit antihyperglycemic effects 31. Based on the findings from previous research highlighting the effectiveness of resveratrol in alleviating liver damage linked to diabetes, we postulated that LR could serve as a promising therapeutic intervention for mitigating diabetes-driven liver alterations in diabetic subjects. Consequently, our goal was to assess the effects of LR on diabetes-caused liver damage.

In type 2 diabetes, insulin resistance leads to enhanced hepatic glucose production and lipid accumulation, culminating in metabolic disruptions and instigating inflammatory reactions. This lipid buildup in the liver instigates the secretion of pro-inflammatory cytokines and activation of inflammation-mediating pathways, intensifying liver inflammation 32. Furthermore, increased blood glucose levels promote the production of advanced glycation end products (AGEs), leading to the elicitation of inflammatory pathways, which further cause the upregulation of various inflammatory markers. For instance, administration of resveratrol for four consecutive months at 5 mg/kg per day strikingly suppressed TNF-α, cyclooxygenase-2, IL-6, and NF-kB in the pancreatic tissues 33. Comparable findings were noted in another study, wherein the expression of proinflammatory markers NF-kB, IL-1β, and IL-6 was heightened, while treatment with resveratrol resulted in a notable decrease in these markers 34. Likewise, the findings of our experimental study closely parallel those of prior research, which demonstrated elevated expression of TNF-α, IL-6, and NF-kB markers in diabetic animals. In an attempt to alleviate inflammation, they were provided LR at doses of 20 mg/kg and 40 mg/kg. Briefly, a notable diminution in the degrees of these proinflammatory indicators was noted in the liver tissues when provided with LR at 40 mg/kg. Although LR at 20 mg/kg showed considerable improvement in liver tissue inflammation, more pronounced and dose-dependent effects were observed at the 40 mg/kg dosage. In contrast, administration of resveratrol alone led to increased expression of these inflammatory markers compared to both doses of LR. In comparison standalone administration of resveratrol at mg/kg, our findings illustrate the effectiveness of LR administration in diminishing inflammation and enhancing the occurrence of diabetic liver damage, particularly at dosages of 20 and 40 mg/kg.

Oxidative stress in diabetes arises from a disparity in reactive oxygen species synthesis and the body's antioxidative defenses. The interplay between diabetes and oxidative stress remains incompletely understood, with only limited knowledge available regarding their connection. Numerous reports have demonstrated that hyperglycemia triggers heightened generation of reactive oxygen species via diverse interconnected links, such as mitochondrial dysfunction, NADPH oxidase activation, and advanced glycation end-products (AGEs) production 35. Due to the elevated production of ROS, oxidative stress induces damage to macromolecules such as lipids, DNA, and proteins by disrupting the levels of antioxidant enzymes 35. Research indicated that diabetic rats display diminished quantities of CAT and GPx, alongside elevated lipid peroxidation. However, the commencement of resveratrol therapy at 20 mg/kg over a span of 10 weeks in diabetic rats notably restored antioxidant enzyme levels while decreasing lipid peroxidation 36. In another experimental study, comparable results were observed, with a 15-day regimen of resveratrol treatment at 10 mg/kg demonstrating a reduction in oxidative stress by restoring catalase, superoxide dismutase, and glutathione levels to their baseline 37. Our data corroborate these results, providing an overview of how reactive oxygen species can inhibit antioxidant defense mechanisms, resulting in reduced activities of antioxidant enzymes. However, the application of LR at 20 and 40 mg/kg notably reduced oxidative stress by rising them, with more pronounced effects observed at the 40 mg/kg dosage. In contrast to LR administered at both administered dosages, a lesser improvement in the levels of these oxidative damage markers was observed with 40 mg/kg resveratrol treatment in diabetic rats. This indicates that both forms of resveratrol, whether LR or resveratrol alone, effectively alleviate oxidative stress induced by hyperglycemia. Hence, they possess the potential to maintain the balance between ROS and the antioxidant defense mechanisms, thereby attenuating the cascade of oxidative damage in the liver of rats with diabetes.

One of the unwanted effects of diabetes on the liver's role involves hyperglycemia, which impairs the enzymatic processes crucial for liver function. Elevated blood sugar levels, insulin resistance, and fat accumulation, all characteristic of diabetes, can hinder the liver's capacity to absorb nutrients and detoxify harmful substances. This impairment can aid in the progression of hepatic diseases in diabetic individuals. As demonstrated in various studies, diabetic rats display raised levels of AST and ALT, indicating liver malfunctioning 38, 39. Although these levels were elevated in rats with diabetes, resveratrol supplementation at 20 mg/kg was seen to normalize the quantities of these enzymes, indicating improvement in liver function in diabetic rats with hyperlipidemia 40. Another study revealed that administering resveratrol to diabetic rats significantly alleviated liver injury by reducing levels of aspartyl aminotransferase, alanine aminotransferase, and bilirubin. These findings underscore the potential of resveratrol in ameliorating liver damage associated with diabetes 37. In this study, our results align with previously reported findings, where diabetic rats exhibited liver damage characterized by alleviated marks of SGOT, SGPT, and GGT. However, administration of LR at both doses demonstrated significant alleviation in the levels of these enzymes, signifying the ameliorative effect of LR in mitigating liver damage. Although both doses were effective in reducing liver damage, more pronounced results were observed in the concentration of 40 mg/kg in contrast to 40 mg/kg of resveratrol independently.

The interplay between oxidative stress, inflammation, and apoptosis in diabetic liver damage is intricate and multifaceted. In a vicious cycle that exacerbates liver damage, ROS can initiate the synthesis of pro-inflammatory cytokines, which in turn can increase ROS production. Oxidative stress and inflammation, both independently and synergistically, lead to apoptosis, resulting in significant hepatocyte loss and liver dysfunction 41. The oxidative stress-induced damage to mitochondria initiates two critical processes in programmed cell death, for instance, the liberation of cytochrome c and the activation of caspases. Similarly, prolonged oxidative stress and inflammation can induce endoplasmic reticulum (ER) stress, further contributing to apoptosis 42. In this study, STZ-induced diabetes in rats resulted in higher expression of apoptotic markers like Bax and caspase 3, along with a reduction in BCL-2 expression in liver tissues. This is consistent with previous reports that indicate elevated levels of caspase 3 and BAX, and decreased BCL-2 expression under diabetic conditions 43, 44. We observed that administering LR at doses of either 20 mg/kg or 40 mg/kg effectively modulated apoptotic markers, indicating reduced liver damage. Although both doses showed promising results, the 40 mg/kg treatment yielded more significant effects compared to the 20 mg/kg dose. Furthermore, a similar modulation of apoptotic markers was observed in the resveratrol-treated group, evidenced by decreased caspase 3 and Bax levels and increased BCL-2 expression. However, these changes were less significant compared to the LR treatment. Our findings fully support previous reports indicating that resveratrol treatment, a natural phytochemical abundant in grapes and berries, alleviates apoptosis in diabetic rats by downregulating caspase 3 and Bax while upregulating the BCL-2 gene 45, 46. As both oxidative stress and inflammation play regulatory roles in apoptotic pathways, our hypothesis suggests that LR's anti-apoptotic effects are primarily attributed to its capacity to diminish oxidative stress and inflammation. Consequently, LR was found to prevent diabetes-associated apoptosis in liver cells.

The notable reduction in liver damage and histological changes can be attributed to LR's distinct anti-inflammatory, antioxidant, and anti-apoptotic properties. Histological examinations revealed significant alterations in STZ-treated diabetic rats, characterized by neutrophil infiltration and pronounced acute and chronic inflammation, indicating the initiation of liver damage associated with diabetes. In comparison to the resveratrol 40 mg/kg dosage, administering LR at 40 mg/kg demonstrated superior efficacy in ameliorating liver damage by mitigating histopathological changes. Additionally, resveratrol therapy was observed to decrease chronic inflammation and improve liver morphology in diabetic animals, consistent with earlier research 47. Thus, while resveratrol alone improved the restoration of normal morphology, as well as biochemical and molecular parameters in diabetic mice, our findings suggest that administering LR was more effective in alleviating hyperglycemia-induced liver damage.

The selection of doses for resveratrol and liposomal resveratrol as well as the sample size number in our study was based on previous research findings. The 20 mg/kg and 40 mg/kg doses of LR were based on our previous investigations, which demonstrated their therapeutic efficacy in various pathological conditions. Specifically, our prior studies 17, 18 established the effectiveness of these doses in mitigating diabetic nephropathy and cardiotoxicity via anti-inflammatory and antioxidant mechanisms. Additionally, previous research, including studies by Alanazi *et al.* and Alhusaini *et al.*, has validated the 20 mg/kg dose in cardioprotective and hepatoprotective models 28, 48. For the free form of resveratrol, we selected the 40 mg/kg dose based on prior studies, such as Kodali *et al.*, which demonstrated significant biological effects at this dose 49. This allowed a direct comparison with the LR formulation at doses previously validated in experimental models. Furthermore, the selection of six rats per group was supported by our previous studies 17, 18, which demonstrated sufficient statistical power to detect significant differences. Our study was conducted in accordance with ethical guidelines and received Institutional Animal Care and Use Committee (IACUC) approval under protocol number KSU-SE-23-36.

Although the promising results, this study has few limitations. First, the research was conducted solely in a rat model, which may not fully replicate the complexity of human diabetic conditions. Further studies including clinical trials, are needed to validate the therapeutic potential of LR in human diabetes. Additionally, while the study investigated various biochemical markers and histopathological changes, it did not address the long-term effects of LR treatment or its safety profile over extended periods. Future research should investigate the molecular mechanisms behind LR's protective effects, as well as potential drug interactions. Exploring LR in combination with other therapeutic agents could also provide valuable insights into its efficacy for treating diabetic liver damage and associated complications. Furthermore, more research into LR's pharmacokinetics and bioavailability would be essential for clinical application.

## 5. Conclusion

In conclusion, we present the first evidence of LR's ability to counteract the molecular, biochemical, and microscopic modifications driven by STZ in hepatic tissue. Our findings indicate that LR significantly affects diabetic hepatic damage by modulating the expression of apoptotic markers, oxidative stress indices, inflammatory mediators, and liver enzymes. This implies that LR may help mitigate hyperglycemia-induced diabetic liver damage and holds the potential for treating diabetic liver damage and other related complications. Next-phase research should probe the precise underlying steps of LR's defensive properties and evaluate its therapeutic potential in clinical settings.
